# Supplementary material for: A disrupted compartment boundary underlies abnormal cardiac patterning and congenital heart defects
Source: Nat Cardiovasc Res. 2025 Dec 29;5(1):67–83. doi: 10.1038/s44161-025-00755-6 (PMC12811143; doi:10.1038/s44161-025-00755-6)
Supplement: Supplementary file 1 — Supplementary Figs. 1–4. [file 44161_2025_755_MOESM1_ESM.pdf]

nature cardiovascular research

Supplementary information https://doi.org/10.1038/s44161-025-00755-6

A disrupted compartment boundary underlies abnormal cardiac patterning and congenital heart defects

In the format provided by the authors and unedited

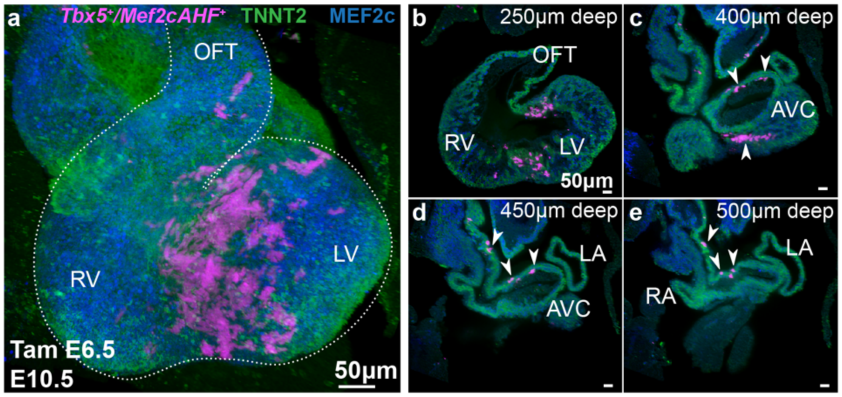

Supplementary Figure 1. *Tbx5⁺/Mef2cAHF⁺* lineage contributions to the developing atria. a-e, In the developing atria at E10.5, optical sections from ventral to dorsal show lineage-labeled *Tbx5⁺/Mef2cAHF⁺* lineage cells marked at E6.5 by a single tamoxifen dose is located from the AV canal (AVC) region to the roof of the atrium, prior to the formation of the interatrial septum, in *Tbx5$^{CreERT2/+}$*;*Mef2cAHF-DreERT2*;*ROSA26$^{Ai6/Ai66}$* embryos. All scale bars equal 50 microns.

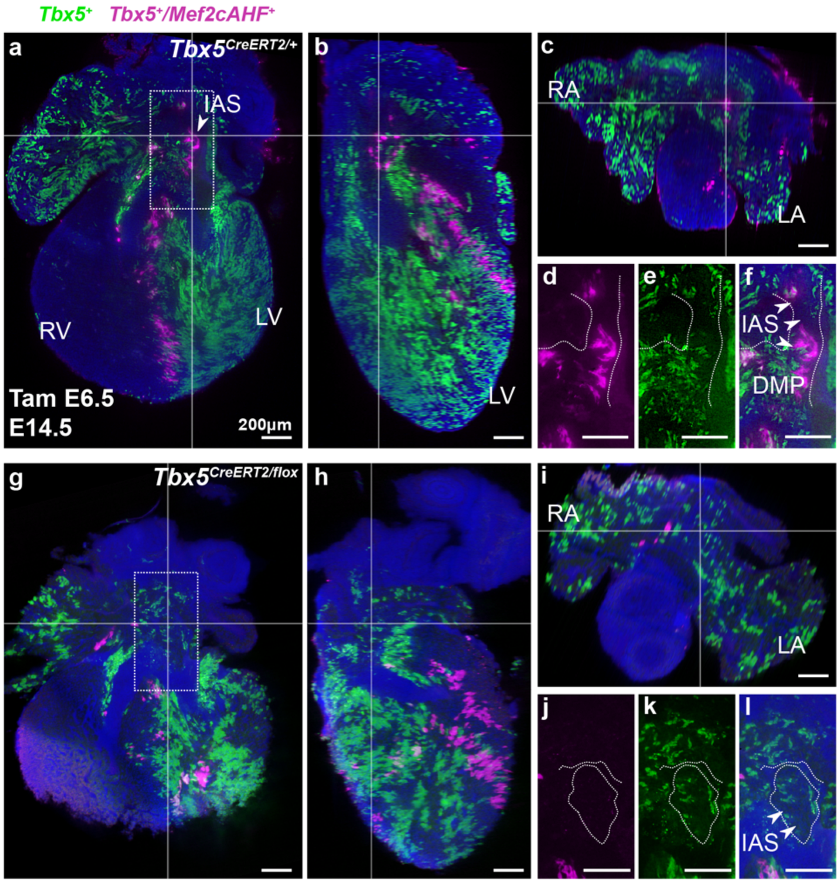

Supplementary Figure 2. Reduced TBX5 dosage leads to reduced contributions of the *Tbx5⁺/Mef2cAHF⁺* lineage to the inter-atrial septum and dorsal mesenchymal protrusion. a-f, At E14.5, the *Tbx5⁺/Mef2cAHF⁺* lineage cells contribute to the interatrial septum (IAS, arrowheads) and dorsal mesenchymal protrusion (DMP) in *Tbx5*$^{CreERT2/+}$ controls (*Tbx5*$^{CreERT2/+}$;*Mef2cAHF-DreERT2*;*ROSA26*$^{Ai6/Ai66}$). **g-l**, The *Tbx5⁺/Mef2cAHF⁺* lineage cells are less apparent in *Tbx5* mutants (*Tbx5*$^{CreERT2/flox}$;*Mef2cAHF-DreERT2*;*ROSA26*$^{Ai6/Ai66}$). Right ventricle (RV), left ventricle (LV), right atrium (RA), left atrium (LA). All scale bars equal 200 microns.

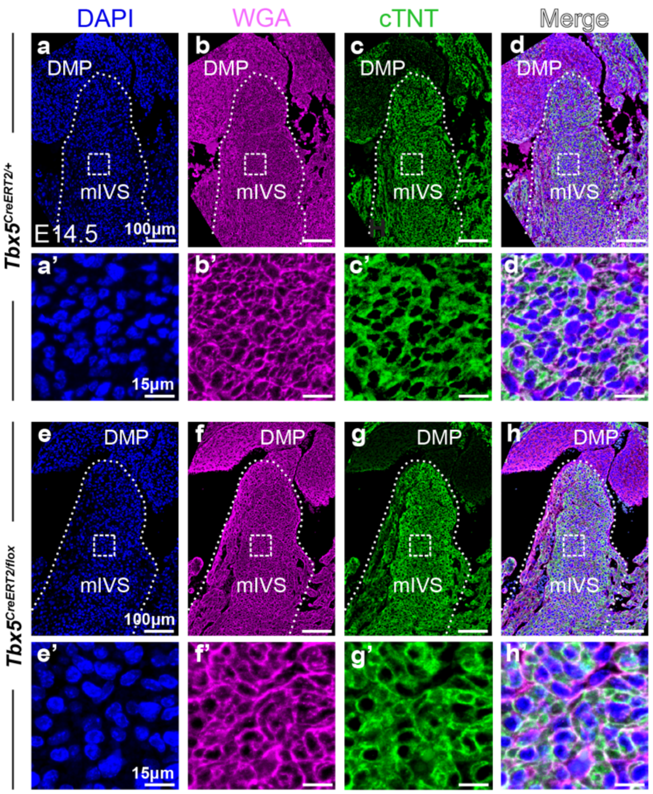

Supplementary Figure 3. Images for quantification of cell geometry, and assessment of tissue architecture in *Tbx5* mutants. a-d', *Tbx5*$^{CreERT2/+}$ control (*Tbx5*$^{CreERT2/+}$;*Mef2cAHF-DreERT2*;*ROSA26*$^{Ai6/Ai66}$) (3 planes per sample, 2 samples) and (**e-h'**) *Tbx5*$^{CreERT2/flox}$ mutant (*Tbx5*$^{CreERT2/flox}$;*Mef2cAHF-DreERT2*;*ROSA26*$^{Ai6/Ai66}$) hearts (3 planes per sample, 2 samples) after single dose of tamoxifen injected at E6.5 is shown at E14.5, stained with DAPI, wheat germ agglutinin (WGA), or cardiac troponin T (cTNT). (b, b', f, f') are repeat images of Figure 4u-v'. scale bars: 100 microns (a-h); 15 microns (a'-h').

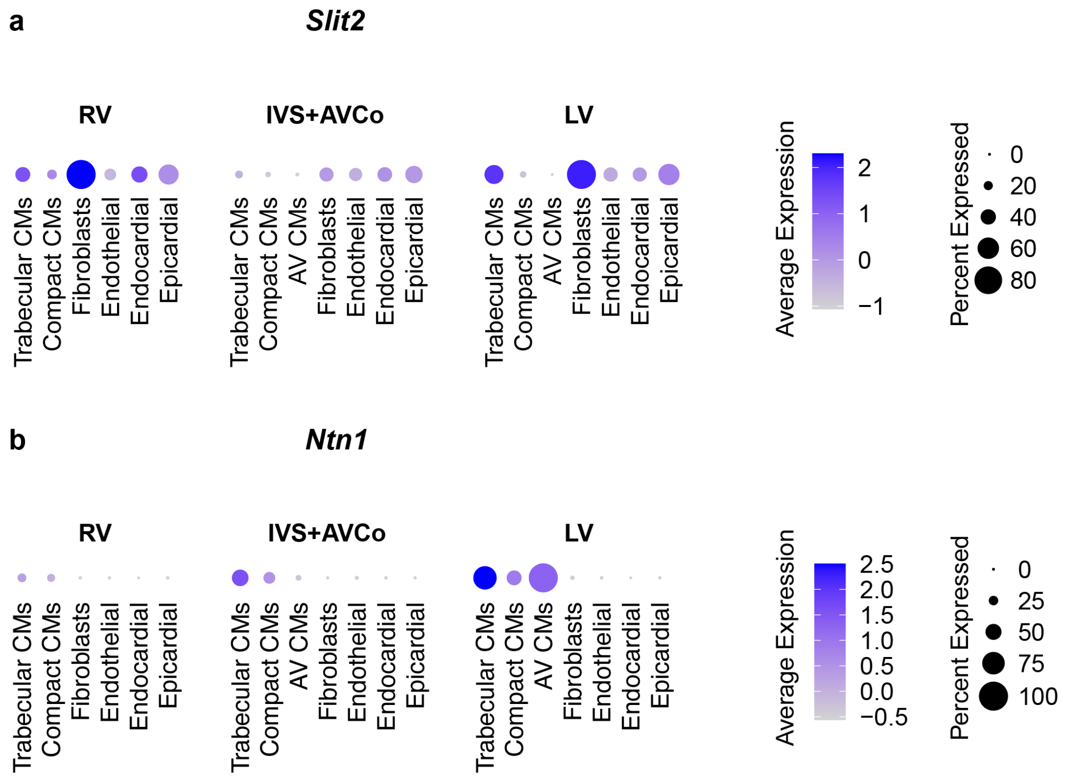

Supplementary Figure 4. Gene expression of *Slit2* and *Ntn1* by cell type in individual cells of the developing heart. By scRNA-seq from control E13.5 hearts (n=4), gene expression of (**a**) *Slit2* and (**b**) *Ntn1* are shown by region (left ventricle (LV), right ventricle (RV), interventricular septum (IVS)+atrioventricular complex (AVCo) regions).
