# Supplementary material for: A disrupted compartment boundary underlies abnormal cardiac patterning and congenital heart defects
Source: Nat Cardiovasc Res. 2025 Dec 29;5(1):67–83. doi: 10.1038/s44161-025-00755-6 (PMC12811143; doi:10.1038/s44161-025-00755-6)
Supplement: Supplementary file 2 — Reporting Summary [file 44161_2025_755_MOESM2_ESM.pdf]

nature portfolio

Corresponding author(s): Kathiriya, Bruneau

Last updated by author(s): October 31, 2025

Reporting Summary

Nature Portfolio wishes to improve the reproducibility of the work that we publish. This form provides structure for consistency and transparency in reporting. For further information on Nature Portfolio policies, see our Editorial Policies and the Editorial Policy Checklist.

Statistics

For all statistical analyses, confirm that the following items are present in the figure legend, table legend, main text, or Methods section.

n/a	Confirmed	
☐	☒	The exact sample size (*n*) for each experimental group/condition, given as a discrete number and unit of measurement
☐	☒	A statement on whether measurements were taken from distinct samples or whether the same sample was measured repeatedly
☐	☒	The statistical test(s) used AND whether they are one- or two-sided<br>*Only common tests should be described solely by name; describe more complex techniques in the Methods section.*
☒	☐	A description of all covariates tested
☐	☒	A description of any assumptions or corrections, such as tests of normality and adjustment for multiple comparisons
☐	☒	A full description of the statistical parameters including central tendency (e.g. means) or other basic estimates (e.g. regression coefficient) AND variation (e.g. standard deviation) or associated estimates of uncertainty (e.g. confidence intervals)
☐	☒	For null hypothesis testing, the test statistic (e.g. F, t, r) with confidence intervals, effect sizes, degrees of freedom and P value noted<br>*Give P values as exact values whenever suitable.*
☒	☐	For Bayesian analysis, information on the choice of priors and Markov chain Monte Carlo settings
☒	☐	For hierarchical and complex designs, identification of the appropriate level for tests and full reporting of outcomes
☒	☐	Estimates of effect sizes (e.g. Cohen's *d*, Pearson's *r*), indicating how they were calculated

Our web collection on statistics for biologists contains articles on many of the points above.

Software and code

Policy information about availability of computer code

Data collection	We used Aperia Versa software with the Leica Versa 200 Automated Slide Scanner to acquire images of serial sections. Zen software with the Zeiss Lightsheet Z.1, additional details for lightsheet image preprocessing is found in Methods; RNAscope image stitching using the Olympus FV3000RS software, Cellranger 2.02 (10X Genomics) for read alignments and Cellaggr for read depth normalization.
Data analysis	Outputs from the Cellranger pipeline were analyzed using the Seurat v3 (Stuart et al., 2019), ImageJ (several plugins are noted in Methods), Code related to “Image Volume Quantification” (Dominguez et al., 2023) is available at https://github.com/mhdominguez/LSFMProcessing. Source data and scripts used to analyze other morphometrics from light sheet microscopy, such as right-left positioning, orientation and directionality of cells, are available at github.com/mhdominguez/Kathiriya-NCVR-2025-analysis. MatLab (Mathworks) for implementing CODA (Kiemen et al., 2022).

For manuscripts utilizing custom algorithms or software that are central to the research but not yet described in published literature, software must be made available to editors and reviewers. We strongly encourage code deposition in a community repository (e.g. GitHub). See the Nature Portfolio guidelines for submitting code & software for further information.

Data

Policy information about availability of data

All manuscripts must include a data availability statement. This statement should provide the following information, where applicable:

- Accession codes, unique identifiers, or web links for publicly available datasets
- A description of any restrictions on data availability
- For clinical datasets or third party data, please ensure that the statement adheres to our policy

Source data is included by Figure. scRNA-seq data generated in this paper is available in the NCBI GEO database (GSE260601). ChIP-seq data from Akerberg et al., 2019, is available in the GEO database (GSE124008).

Research involving human participants, their data, or biological material

Policy information about studies with human participants or human data. See also policy information about sex, gender (identity/presentation), and sexual orientation and race, ethnicity and racism.

Reporting on sex and gender	This study did not involve human subjects.
Reporting on race, ethnicity, or other socially relevant groupings	*Please specify the socially constructed or socially relevant categorization variable(s) used in your manuscript and explain why they were used. Please note that such variables should not be used as proxies for other socially constructed/relevant variables (for example, race or ethnicity should not be used as a proxy for socioeconomic status).*<br>*Provide clear definitions of the relevant terms used, how they were provided (by the participants/respondents, the researchers, or third parties), and the method(s) used to classify people into the different categories (e.g. self-report, census or administrative data, social media data, etc.)*<br>*Please provide details about how you controlled for confounding variables in your analyses.*
Population characteristics	*Describe the covariate-relevant population characteristics of the human research participants (e.g. age, genotypic information, past and current diagnosis and treatment categories). If you filled out the behavioural & social sciences study design questions and have nothing to add here, write "See above."*
Recruitment	*Describe how participants were recruited. Outline any potential self-selection bias or other biases that may be present and how these are likely to impact results.*
Ethics oversight	*Identify the organization(s) that approved the study protocol.*

Note that full information on the approval of the study protocol must also be provided in the manuscript.

Field-specific reporting

Please select the one below that is the best fit for your research. If you are not sure, read the appropriate sections before making your selection.

☒ Life sciences ☐ Behavioural & social sciences ☐ Ecological, evolutionary & environmental sciences

For a reference copy of the document with all sections, see nature.com/documents/nr-reporting-summary-flat.pdf

Life sciences study design

All studies must disclose on these points even when the disclosure is negative.

Sample size	No sample size calculations were performed. Duplicates were considered sufficient for statistical robustness in most cases. When feasible, additional samples were collected and analyzed.
Data exclusions	One Tbx5 mutant sample of the IVS+AVC was not included because it was lost from a microfluidic clog during sample processing.
Replication	Samples were allocated based on embryonic stage or genotype. Multiple replicates and orthogonal experiments, such as fluorescence in situ hybridization, were used to verify the reproducibility of the scRNAseq. Representative images are shown based on experiments that were repeated independently with similar results, as follows: Figure(s) 1a-f: 2 samples; 1g-j: 5 samples; 2a-i: 4 samples; 2j-n: 6 samples; 2o-q: 8 samples; 2r-t: 15 samples; 3a,b: 2 controls, 2 DTA mutants; 3c-j: 2 controls, 4 DTA mutants; 3l-n' and Extended Data 2: 3 controls, 2 DTA mutants; 4a-d: 5 controls and 4 Tbx5 mutants, for which some samples are shown in Extended Data Figure 4a-i; Figure 4i-m: 4 controls, 4n-r: 3 Tbx5 mutants; 4u-v'; 2 controls, 2 Tbx5 mutants; 5i-n; 2 controls and 2 Tbx5 mutants, for which the second replicates are shown in Extended Data Figure 6i-n"; Figure 6f-h: 14 controls, 8 Slit2+/-, 4 Slit2-/-; 6j-l: 14 controls, 9 Ntn1+/-, 7 Ntn1-/-, with some samples shown in Extended Data Figure 10a-d; Extended Data Figure(s) 1: 2 samples per condition; 4a-l: 5 controls, 4 Tbx5 mutants, with some samples shown in Figure 4; 3: 3 controls, 7 Tbx5 mutants; 6a-h': 2 controls and 2 Tbx5 mutants; 6o: 2 controls; 7a-h: 2 controls and 2 Tbx5 mutants; 9a-d: 14 controls, 8 Tbx5+/-, 8 Slit2+/-; 8 Tbx5+/-;Slit2+/-; 9f-i: 14 controls, 7 Tbx5+/-; 9 Ntn1+/-, 7 Ntn1-/-. Supplemental Figure(s) 1: 5 samples; 2: 5 controls, 4 Tbx5 mutants; 3: 2 controls, 2 Tbx5 mutants. Number of replicates are denoted for each graph in figure legends. Several limitations of this study should be considered. Using inducible genetic tracing, there is an inherent variability from recombination of the fluorescent reporters within samples. For some comparisons, more samples of each genotype could have mitigated this limitation, but obtaining additional samples have been technically challenging, in large part due to a requirement of 5 alleles to be present. Notwithstanding, we have tried to account for recombination efficiency, when possible, in imaging analyses. We have made great effort to mitigate this concern by not quantifying

measurements that would be greatly skewed by recombination efficiency. For this reason, for example, we have refrained from commenting on whether a given lineage is quantitatively increased or decreased if fewer samples were available. Instead, we have focused our analyses on the location of the cells, as we feel that this is less affected by recombination efficiency. Using orthogonal approaches, we measure in an unbiased and statistically robust fashion using various metrics of quantitative morphometry from lightsheet or histology images. Our interpretations are based on statistical robustness from a large number of sampled regions per embryo, in the comparisons that are presented. The repeated measurements are akin to the (much larger) measurements in single cell RNAseq.

Randomization	Randomization was not relevant to the design of this study.
Blinding	Blinding was used for qualitative assessment and quantitative morphometry.

Reporting for specific materials, systems and methods

We require information from authors about some types of materials, experimental systems and methods used in many studies. Here, indicate whether each material, system or method listed is relevant to your study. If you are not sure if a list item applies to your research, read the appropriate section before selecting a response.

Materials & experimental systems

n/a	Involved in the study	
☐	☒	Antibodies
☒	☐	Eukaryotic cell lines
☒	☐	Palaeontology and archaeology
☐	☒	Animals and other organisms
☒	☐	Clinical data
☒	☐	Dual use research of concern
☒	☐	Plants

Methods

n/a	Involved in the study	
☒	☐	ChIP-seq
☒	☐	Flow cytometry
☒	☐	MRI-based neuroimaging

Antibodies

Antibodies used	Primary antibodies used were: tdTomato (rabbit, Rockland 600-401-379, 1:1000), MEF2c (sheep, R&D AF6786, 1:250)(Dominguez et al., 2023), TNNT2 (mouse, Thermo MS-295-P, 1:500)(Kathiriya et al., 2021), Netrin-1 antibody 1:500 (R&D Systems AF1109) (Ramkhelawon et al., 2014).
Validation	Selection of antibodies were based on validation in previous publications.

Animals and other research organisms

Policy information about studies involving animals; ARRIVE guidelines recommended for reporting animal research, and Sex and Gender in Research

Laboratory animals	Mice were housed in a barrier animal facility with standard husbandry conditions (dark/light cycle, ambient temperature and humidity) at the Gladstone Institutes. Mice of Tbx5CreERT2IRES2xFLAG (abbreviated here as Tbx5CreERT2) and Mef2cAHF-DreERT2 25, Tbx5del/+ and Tbx5flox/+ 45, ROSA26Ai66 and ROSA26Ai6 35 were described previously. Mef2cAHF-Cre mice 46 were obtained from Brian Black (University of California, San Francisco). Slit2+/- mice (MMRC, Strain 065588-UCD, donated by Kent Lloyd, UC Davis) were generated by CRISPR/Cas9-targeted constitutive deletion of exon 8 and flanking splicing regions of Slit2. Ntn1+/- mice were derived from matings of Ntn1 floxed mice (Ntn1flox/+; Jackson Laboratory #028038) 56 to beta-actin-Cre 57, which were obtained from Gail Martin (University of California, San Francisco). All mouse strains were maintained in the C57BL6/J background (Jackson Laboratory #664), except for Tbx5del/+, which was maintained in Black Swiss (Charles River, Strain Code 492), and Slit2+/- which was maintained in C57BL6/N (Jackson Laboratory, #005304). Both male and female embryos were collected from timed matings and used at random for experiments. We generated an attenuated diptheria toxin (DTA176) transgenic knock-in mouse under the control of the dual-recombinase intersectional cassette. Figure(s) 1a-f: Embryonic day (E) 14.5; 1g-j: E14.5; 2a-i: E8.0; 2j-n: E8.25; 2o-q: E8.5 2r-t: E10.5; 3a,b: E9.5; 3c-j: E12.5; 3l-n' and Extended Data 2: E10.5; 4a-d: E14.5; Figure 4i-m: E14.5, 4n-r: 3E14.5; 4u-v'; 2E14.5; 5i-n; E14.5; Figure 6f-h: E14.5; Extended Data Figure(s) 1: E12.5 (a), E13.5 (b), E14.5 (c), E7.75 (d), E8.0 (e), E8.5 (f); 3: E14.5; 4a-l: E14.5; 5: E13.5, 6a-h': E14.5; 6o: E14.5; 7a-h: E11.5; 9a-d: E14.5; 9f-i: E14.5 Supplemental Figure(s) 1: E10.5; 2: E14.5; 3: E14.5.
Wild animals	The study did not involve wild animals.
Reporting on sex	Sex-based information was not collected for embryonic specimens.
Field-collected samples	The study did not involve samples collected in the field.
Ethics oversight	All mouse protocols were approved (AN203375-00H and AN199784-00E) by the Institutional Animal Care and Use Committee at UCSF.

Note that full information on the approval of the study protocol must also be provided in the manuscript.

Plants

Seed stocks

The study does not involve plants.

Novel plant genotypes

Describe the methods by which all novel plant genotypes were produced. This includes those generated by transgenic approaches, gene editing, chemical/radiation-based mutagenesis and hybridization. For transgenic lines, describe the transformation method, the number of independent lines analyzed and the generation upon which experiments were performed. For gene-edited lines, describe the editor used, the endogenous sequence targeted for editing, the targeting guide RNA sequence (if applicable) and how the editor was applied.

Authentication

Describe any authentication procedures for each seed stock used or novel genotype generated. Describe any experiments used to assess the effect of a mutation and, where applicable, how potential secondary effects (e.g. second site T-DNA insertions, mosiacism, off-target gene editing) were examined.
